# Intrahepatic Biliary Stricture Following Carbon-Ion Radiotherapy: A Narrative Review

**DOI:** 10.7759/cureus.105676

**Published:** 2026-03-22

**Authors:** Hideo Kidogawa, Takahito Tagami, Takeshi Konno, Takashi Okimoto, Nobutaka Matayoshi, Toshihito Uehara, Junya Noguchi, Shin Shinyama, Masao Inoue, Takatomo Yamayoshi, Kohji Okamoto

**Affiliations:** 1 Department of Surgery, Kitakyushu City Yahata Hospital, Fukuoka, JPN

**Keywords:** carbon-ion radiotherapy, dosimetric constraints, hepatocellular carcinoma, intrahepatic biliary stricture, late radiation toxicity

## Abstract

We conducted a narrative review to synthesize the limited and fragmented evidence on intrahepatic biliary strictures following carbon-ion radiotherapy (CIRT) for liver tumors, with a focus on biliary-specific risks amid CIRT's advantages in dose conformity, particularly given the current scarcity of direct clinical data. A focused narrative literature search was performed across academic databases to evaluate study designs, patient populations, and biliary complications. Due to the high clinical heterogeneity and limited sample sizes, we conducted a conceptual qualitative synthesis utilizing a stratified synthesis approach rather than a quantitative meta-analysis. Proxy populations such as those with pancreatic cancer and conventional photon therapy data were referenced selectively to contextualize radiation-related biliary injury, while strictly not considering them as direct evidence.

Intrahepatic biliary stricture was observed as a late-onset complication. Notably, one cohort reported an incidence of 11% (five out of 47 patients) in a specific subgroup with unresectable hepatocellular carcinoma (HCC) and Child-Pugh B cirrhosis, although this finding has not been consistently reproduced across broader populations. Key risk factors identified included perihilar-type tumors, macrovascular invasion, and prior perihilar treatments. Additionally, susceptibility appeared to be elevated by high radiation doses such as biliary V10% ≥ 40 Gy (RBE) and tumor proximity to major biliary structures. For mild cases, conservative care was typically utilized, whereas percutaneous drainage or stenting was required for severe instances. Biliary stricture contributed to liver function deterioration in 23% (one of five) of affected patients in the aforementioned cohort. While many cohorts report manageable toxicities, rare but fatal outcomes such as a 1-2% incidence of grade 5 toxicities, including uncontrollable cholangitis and liver failure, have been documented across broader studies.

Overall, while CIRT demonstrates a generally acceptable safety profile, it is imperative to acknowledge that rare, severe ischemic strictures can precipitate fatal infectious cascades or liver failure. Careful monitoring and adherence to dosimetric constraints are warranted in high-risk patients.

## Introduction and background

Hepatocellular carcinoma (HCC) and intrahepatic cholangiocarcinoma represent major global health burdens, particularly in patients with underlying liver cirrhosis or viral hepatitis [1,2]. In many of these cases, surgical resection or transplantation is infeasible due to tumor location, impaired liver function, or comorbidities [3]. Carbon-ion radiotherapy (CIRT), a form of particle therapy utilizing accelerated carbon ions, has emerged as a promising alternative for localized, unresectable liver tumors [4]. CIRT leverages the Bragg peak phenomenon for precise dose deposition, thereby sparing surrounding healthy tissues while delivering high relative biological effectiveness (RBE) against radioresistant hypoxic regions [1,5].

This approach minimizes exposure to critical structures like the biliary tract, potentially reducing complications such as radiation-induced liver disease when compared to conventional photon-based radiotherapy [6,7]. However, when adjacent hepatobiliary structures are inevitably irradiated due to tumor proximity, radiation-induced biliary strictures can occur. Pathophysiologically, this complication typically arises from microvascular ischemia and progressive fibrosis of the ductal endothelium, potentially culminating in severe clinical consequences such as refractory obstructive jaundice, recurrent cholangitis, and life-threatening liver failure [8]. While the biliary toxicity profiles and tolerance doses of conventional photon-based stereotactic body radiotherapy (SBRT) and proton therapy have been increasingly characterized in recent years, the specific biliary risks associated with CIRT - which uniquely combines steep dose gradients with high RBE - remain less well-defined.

Despite CIRT's clinical adoption since the 1990s, particularly in Japan [5], systematic insights into intrahepatic biliary stricture, defined as a narrowing of the bile ducts within the liver parenchyma following radiation exposure, remain limited. Early studies emphasized CIRT's efficacy in achieving high local control rates; e.g., over 90% in early-stage HCC, with acceptable toxicities [9,10]. Yet, biliary-specific adverse events were often underreported and were overshadowed by broader metrics like overall survival and general hepatotoxicity [3,7]. Furthermore, variations in patient populations, such as those with Child-Pugh B cirrhosis or macrovascular invasion, and treatment parameters like hypofractionated dosing, for example, 60 Gy in four fractions, complicate risk assessment [8,11]. Therefore, we synthesized evidence on the incidence, risk factors, management, and outcomes of intrahepatic biliary stricture following CIRT for liver malignancies. By integrating findings from clinical trials, cohort studies, and reviews, we aimed to clarify its clinical significance and guide future therapeutic strategies.

## Review

Methods

*Study Design* 

Given the scarcity and heterogeneity of primary literature focusing exclusively on CIRT-induced biliary strictures in HCC, we adopted a narrative review approach. This methodological choice enabled a comprehensive qualitative synthesis of available evidence, because the current data structure, characterized by small cohorts and variable follow-up periods, precludes a rigorous quantitative meta-analysis. Importantly, to enrich our mechanistic and dosimetric discussions, we purposefully adopted a stratified synthesis approach. Under this framework, we broadened our inclusion criteria to encompass proxy populations (e.g., pancreatic and cholangiocarcinoma) and alternative radiation modalities (e.g., volumetric modulated arc therapy (VMAT)). We explicitly utilized these proxy studies not to inflate HCC statistics, but strictly as indirect, comparative references to better understand radiation-induced biliary toxicity pathways and to contextualize CIRT's sharp dose fall-off, ensuring they remained distinct from direct HCC evidence.

Search Strategy

A comprehensive search was performed using the Semantic Scholar and OpenAlex databases. These academic graph databases were selected because they automatically ingest and fully index records from primary biomedical databases, including PubMed and MEDLINE, thereby ensuring robust coverage of relevant literature. The search strategy employed a hybrid semantic and keyword-based retrieval approach to maximize coverage.

The following search query combinations were employed: "intrahepatic biliary stricture" AND "carbon-ion radiotherapy" AND ("liver cancer" OR "liver tumor") AND ("complication" OR "radiation-induced cholestasis"); "carbon-ion therapy" AND "biliary tract injury" AND ("radiation complications" OR "intrahepatic stricture" OR "hepatobiliary"); ("particle beam radiotherapy" OR "carbon-ion radiotherapy") AND "hepatocellular carcinoma" AND ("biliary stricture" OR "proton therapy") AND "complication"; "radiation-induced biliary damage" AND ("ion therapy" OR "heavy-ion" OR "carbon beam") AND ("liver irradiation" OR "stricture"); "carbon-ion radiotherapy" AND "liver tumors" AND ("biliary complications" OR "clinical outcomes" OR "dose-volume histogram"); "risk factors" AND "intrahepatic stricture" AND ("carbon-ion treatment" OR "liver dosimetry" OR "biliary obstruction")

Study Selection

**Figure 1:**
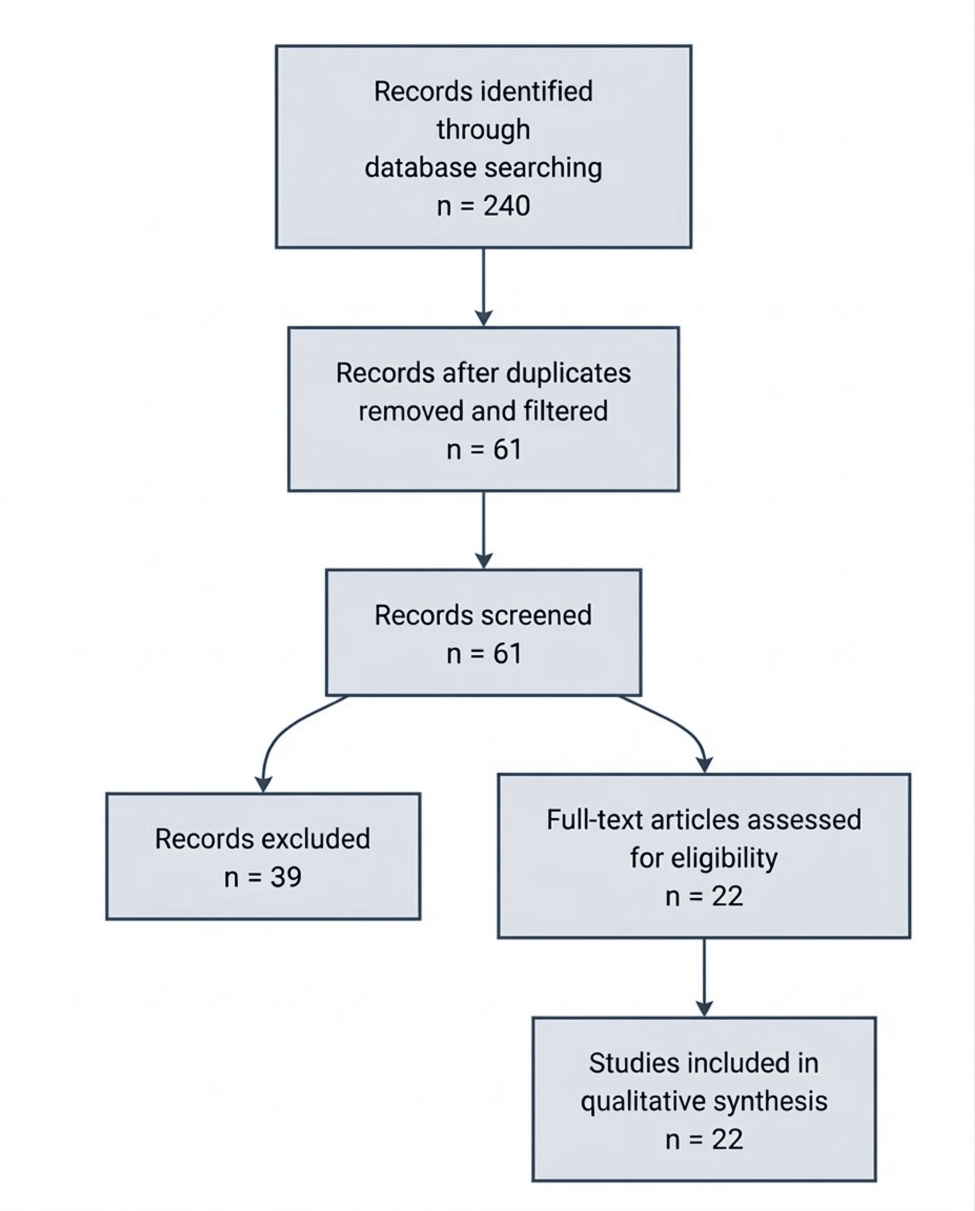
PRISMA flow diagram* depicting the selection of studies ^*^[12] Notably, no studies were excluded at the full-text assessment stage PRISMA: Preferred Reporting Items for Systematic reviews and Meta-Analyses

The initial database search identified 240 records. Following the removal of duplicates and relevance-based filtering, 61 records were screened against predefined eligibility criteria. Of these, 39 papers were excluded, leaving 22 papers for inclusion in the final qualitative synthesis. The detailed selection process is illustrated in the PRISMA (Preferred Reporting Items for Systematic reviews and Meta-Analyses) flow diagram in Figure 1. Because this study was designed as a narrative review, the PRISMA-style flow diagram is presented for transparency only and does not represent a formal systematic review process. The PRISMA guidelines are open-access and free to use.

Eligibility criteria required that the included studies involve human participants, explicitly excluding animal models or in vitro experiments. Furthermore, the studies had to specifically investigate CIRT for liver or hepatobiliary conditions and report on intrahepatic biliary stricture or related biliary complications as an outcome. We also required that the papers be clinically relevant, such as clinical reports, case series, trials, or reviews, and that they involve patient outcomes rather than purely dosimetric simulations. Additionally, the studies needed to primarily include patients treated for liver tumors, such as HCC or hepatic metastases, and address post-treatment complications occurring within one to five years after radiotherapy, such as stricture development. Finally, eligible records had to discuss risk factors, incidence rates, or mechanisms of radiation-induced biliary injury, or describe aspects such as the diagnosis, treatment, or prognosis of biliary strictures following CIRT.

To maintain transparency and comprehensiveness where direct evidence was sparse, we applied explicit criteria under our stratified synthesis approach to retain certain studies that did not meet the primary HCC-CIRT eligibility criteria. For instance, studies focusing primarily on intrahepatic cholangiocarcinoma or oligometastatic liver disease [2,13,14] were included; these provided direct evidence on biliary complications in comparable hepatobiliary contexts following CIRT. Conversely, proxy populations and alternative modalities were systematically separated and retained strictly for indirect mechanistic insights. Specifically, studies examining pancreatic cancer populations [15,16,17,18] and nasopharyngeal carcinoma [19] were included exclusively to provide contextual parallels regarding CIRT toxicities near adjacent critical structures. Finally, a study utilizing VMAT rather than CIRT [6] was retained solely for comparative risk factor analysis. Importantly, data from these proxy studies were deliberately excluded from our primary incidence calculations for HCC.

Quality Assessment

Although we did not employ a formal risk-of-bias assessment tool (e.g., ROBINS-I or Cochrane RoB 2) due to the narrative design of this review, we actively mitigated this limitation by implementing a rigorous qualitative "Confidence Level" assessment. For each synthesized theme and included study, we evaluated the methodological quality based on study design and sample size, ensuring transparency regarding the strength and reliability of the underlying evidence.

Data Extraction and Synthesis

Data extraction focused on the following variables:

Study design: The study type (e.g., case report, clinical trial, retrospective cohort), sample size, and duration of follow-up were extracted.

Patient population: Patient characteristics, including the number of patients, primary diagnosis (e.g., hepatocellular carcinoma), and relevant comorbidities, were recorded.

Treatment details: Carbon-ion radiotherapy parameters, such as total dose, fractionation, and target areas (e.g., liver tumors), were specified.

Biliary complications: The occurrence of intrahepatic biliary stricture, including the incidence rate, timing post-treatment, and diagnostic methods, was detailed.

Risk factors: Reported risk factors for developing biliary stricture, such as radiation dose to biliary structures, tumor location, or prior treatments, were identified.

Outcomes and management: Clinical outcomes, including the severity of strictures, management approaches (e.g., stenting, surgery), and long-term prognosis, were summarized.

Following the extraction of these variables, we synthesized the key findings using our predefined stratified approach. Data from primary hepatobiliary cohorts were prioritized to evaluate direct safety and efficacy profiles, whereas data extracted from proxy populations were synthesized separately to strictly inform indirect mechanistic discussions. Thematic analysis was employed to identify patterns and synthesize findings across the included studies. Because a formal risk-of-bias tool was not utilized, evidence strength was qualitatively assessed by assigning a "Confidence Level" (e.g., high, moderate, or low) to each synthesized theme. This assessment was rigorously based on study design, sample size, and the overall consistency of the findings.

Results

Characteristics of the Included Studies

The included studies spanned from 2005 to 2025, and were predominantly characterized as retrospective cohorts and reviews focused on HCC or related hepatobiliary malignancies (Table 1). Sample sizes ranged from single cases, such as those involving transplanted livers [20], to over 100 patients, and included broad narrative syntheses [21]. Treatment regimens emphasized hypofractionated CIRT (e.g., 60 Gy (RBE) in four fractions) targeting unresectable tumors. In accordance with our stratified synthesis approach, proxy populations (e.g., those with pancreatic or metastatic disease) were referenced selectively to contextualize radiation-related biliary injury, while being strictly excluded from primary HCC incidence calculations and not considered direct evidence.

**Table 1:** Characteristics of the included studies ^*^Under our stratified synthesis approach, proxy populations (e.g., cholangiocarcinoma, pancreatic cancer, nasopharyngeal carcinoma, oligometastatic liver disease) were included to provide indirect mechanistic parallels or comparative risk factor analyses, as direct HCC/CIRT evidence is sparse. Data from these populations were deliberately excluded from primary HCC incidence calculations and should be interpreted strictly as contextual evidence. ^**^This study utilized VMAT rather than CIRT and was retained exclusively for comparative risk factor analysis HCC: hepatocellular carcinoma; CIRT: carbon-ion radiotherapy; RBE: relative biological effectiveness; VMAT: volumetric modulated arc therapy; LET: linear energy transfer; PBS: pencil beam scanning

Study	Study type	Sample size	Population	Treatment details	Key focus
Abousaida et al. (2021) [1]	Narrative review	Not applicable	Localized unresectable HCC	CIRT with superior dose distribution	Dosimetric advantages for HCC
Kasuya et al. (2019) [2]	Retrospective cohort	56	Unresectable cholangiocarcinoma^*^	76 Gy (RBE) in 20 fractions	Survival and toxicity in cholangiocarcinoma
Byun et al. (2023) [3]	Review	Synthesis	HCC	CIRT with Bragg peak	Clinical outcomes
Choi et al. (2024) [4]	Narrative review	Not applicable	Various malignancies, including HCC	CIRT with high RBE	Clinical indications
Tsujii and Kamada (2012) [5]	Review	Synthesis	Various solid tumors, including the liver	CIRT with optimized dosing	Clinical results
Nassar et al. (2019) [6]	Prospective cohort	25	Inoperable HCC	50.4 Gy in 28 fractions (VMAT)^**^	Radiation-induced liver damage
Yun et al. (2024) [7]	Systematic review	Synthesis (34 studies)	Various solid tumors, including the liver	CIRT vs. conventional	Effectiveness and safety
Maki et al. (2025) [8]	Retrospective cohort	103	HCC with perihilar or portal vein involvement	60 Gy in 4 fractions	Adverse events and biliary stricture risk
Kato et al. (2005) [9]	Phase I/II trial	Not specified	HCC	2-fraction CIRT	Preliminary safety
Shibuya et al. (2021)[10]	Prospective study	24 (enrolled 35)	HCC ≤ 10 cm, no vascular invasion	52.8 or 60 Gy (RBE) in 4 fractions	Safety and efficacy
Hiroshima et al. (2023) [11]	Retrospective cohort	47	Unresectable HCC with Child-Pugh B	52.8-60 Gy (RBE) in 4-12 fractions	Toxicity in Child-Pugh B HCC
Mizumoto et al. (2024) [13]	Prospective registry and meta-analysis	85 (26 CIRT)	Intrahepatic cholangiocarcinoma^*^	Particle therapy (CIRT subset)	Efficacy and toxicity in ICC
Shiba et al. (2023) [14]	Retrospective multicentric	102 (121 tumors)	Oligometastatic liver disease^*^	58-76 Gy (RBE) in 1-20 fractions	Outcomes in oligometastases
Malouff et al. (2020) [15]	Narrative review	Synthesis from early studies	Pancreatic cancer^*^	CIRT with high LET	Role in pancreatic cancer
Okamoto et al. (2021) [16]	Prospective evaluation	10	Recurrent unresectable pancreatic cancer^*^	55.2 Gy (RBE) in 12 fractions	Feasibility of re-irradiation
Shinoto et al. (2012) [17]	Phase 1 trial	Not specified	Resectable pancreatic cancer^*^	Short-course preoperative CIRT	Tolerance and efficacy
Yamamoto et al. (2023) [18]	Retrospective comparative	35 CIRT, 110 standard	Resectable pancreatic cancer^*^	CIRT monotherapy	Comparison with standard therapy
Hu et al. (2020) [19]	Retrospective cohort	43	Recurrent nasopharyngeal carcinoma^*^	57.6-65 Gy (RBE) in 12-16 fractions	Outcomes in re-irradiation
Hayashi et al. (2025) [20]	Case report	1	HCC in a transplanted liver	60 Gy (RBE) in 4 fractions	Safety of the transplanted liver
Meyer et al. (2007) [21]	Narrative review	Synthesis	Gastrointestinal malignancies, including liver	Particle therapy, including CIRT	Physics and clinical experience
Zhang et al. (2023) [22]	Retrospective cohort	90	HCC	Pencil beam scanning CIRT	Efficacy and toxicity of PBS CIRT
Kidogawa et al. (2026) [23]	Case report	1	HCC near the hepatic hilum	CIRT for a centrally located tumor	Fatal infectious cascade following refractory radiation-induced biliary stenosis

Thematic Findings

Incidence and timing of intrahepatic biliary stricture: Intrahepatic biliary stricture following CIRT was observed as a late complication with a low to moderate incidence. Specifically, it was noted in 11% of patients (five out of 47) with unresectable HCC and Child-Pugh B cirrhosis at a median onset of eight months post-treatment, notably with zero treatment-related deaths reported in this cohort [11]. Conversely, in a broader cohort using pencil-beam scanning CIRT, the incidence was 2.2% (two of 90 patients). These variations indicate that, although overall incidence is low, susceptibility is strongly influenced by anatomical and patient-specific factors. CT or MRI demonstrated proximal biliary dilation, and diagnoses were subsequently confirmed by elevated liver enzymes or clinical signs of cholangitis.

In contrast, isolated grade 3 bile duct stenosis was reported in only one case of intrahepatic cholangiocarcinoma among a cohort of 56 patients [2], with no other severe biliary events noted across broader cohorts [14]. The timing of onset varied widely; strictures emerged over an extended period in HCC patients, distinguishing them from more predictable acute adverse events. Conversely, no grade 3 or higher biliary toxicities were reported in prospective hypofractionated studies involving 24 HCC patients who were followed for a median of 25 months, which similarly reported zero treatment-related deaths [10]. While diagnostic consistency was maintained through the use of imaging and biochemical markers, direct comparability was limited by variations in follow-up durations (e.g., a median of 14.8 months in cholangiocarcinoma versus 25 months in Child-Pugh B cohorts). Consequently, shorter follow-up periods may have underestimated the incidence of late-onset cases.

Risk factors for biliary stricture development: Key risk factors for intrahepatic biliary stricture following CIRT were identified across the included studies. Tumor-related features, such as perihilar-type HCC, involvement of the portal vein trunk branch area, and macrovascular invasion, alongside prior perihilar treatments, were associated with high-grade adverse events in a cohort of 103 HCC patients [8]. Furthermore, dosimetric factors - specifically high radiation exposure (defined as biliary V10% ≥ 40 Gy (RBE) in four fractions, corresponding to an EQD2 of approximately 104 Gy assuming an α/β ratio of 3 for biliary tissues) - were found to independently predict stricture development through multivariate analysis in a single retrospective Child-Pugh B cohort [11], alongside tumor adjacency to major biliary structures. Among the five affected patients, prior transarterial chemoembolization (TACE) was identified as a risk factor in three cases. Conversely, no correlation was found between stricture development and baseline Child-Pugh scores or tumor sizes.

In reviews comparing modalities, CIRT's sharp dose fall-off was highlighted as a mitigating factor for biliary injury when compared to conventional photon therapy [7]. Interestingly, overarching risk factors were not definitively identified in prospective hypofractionated studies or broader cholangiocarcinoma cohorts [2,10]. However, it is noteworthy that an isolated case of grade 3 stenosis with ascites was reported following a prescription of 76 Gy (RBE) in 20 fractions (EQD2 (α/β=3) of approximately 103 Gy) [2]. This general absence of reported factors may be attributable to strict exclusion criteria, such as the requirement of > 1 cm separation from the alimentary tract. Finally, discrepancies were observed in metastatic contexts; no biliary risks were specified despite high dosing regimens (58-76 Gy (RBE)). This was attributed to differences in primary tumor origins and shorter median follow-up periods (e.g., 19 months) compared to primary HCC cohorts [14].

Management and clinical outcomes of biliary strictures: Conservative supportive care was the primary approach for mild (grade 2) cases. Conversely, interventional approaches, such as percutaneous transhepatic biliary drainage (PTBD) or stenting, were employed for severe instances. Specifically, these interventions were required for three of five affected patients in the Child-Pugh B cohort [11]. This complication was observed to lead to liver function deterioration in 23% of affected patients, although no treatment-related mortality was reported. In terms of broader outcomes, strictures were noted to contribute to the progression to Child-Pugh C cirrhosis in 40% of these cases. Despite these biliary complications, overall clinical outcomes remained favorable; a two-year overall survival rate of 68% and an 85% one-year local control rate were achieved, underscoring CIRT's efficacy [11].

No specific management details were reported in cases of isolated stenosis or within prospective hypofractionated regimens, where grade 3 toxicities were completely absent. It was suggested that early detection via prolonged imaging surveillance might mitigate the severity of these complications. Furthermore, the measurement of outcomes was found to vary across studies. For instance, the Common Terminology Criteria for Adverse Events (CTCAE) grading was strictly applied in HCC cohorts, whereas more general toxicity reporting was utilized in proxy populations. This methodological variation was considered to potentially inflate the perceived mildness of complications in studies with shorter follow-up periods.

Crucially, while some specific cohorts reported no treatment-related mortality, broader literature indicates that CIRT can lead to rare but fatal (grade 5) biliary toxicities. Specifically, Maki et al. reported grade 5 biliary strictures in two of 103 patients (1.9%), with both deaths attributable to refractory cholangitis despite endoscopic stenting [8]. Similarly, Zhang et al. documented one fatal case in which recurrent jaundice following a bile duct stricture led to death 25 months post-treatment [22]. Furthermore, Kasuya et al. reported four deaths due to liver failure among 56 cholangiocarcinoma patients (7.1%); two of these were associated with persistent cholangitis and biliary stricture, although a direct causal relationship with CIRT could not be definitively established in these cases [2]. Aligning with these findings, a recent case report by the present author, serving strictly as anecdotal evidence, detailed a fatal infectious cascade driven by emerging opportunistic pathogens following refractory radiation-induced biliary stenosis [23]. These instances emphasize that while the overall incidence is low, the consequences of severe ischemic strictures can be lethal.

Dosimetric and biological advantages mitigating biliary risks: The physical attributes of CIRT, including its sharp dose fall-off and high linear energy transfer (LET), have been reported to minimize biliary exposure compared with conventional radiotherapy. Consequently, CIRT represents an optimal modality for HCC located near critical structures, with no direct stricture reports identified in narrative syntheses [1,5].

Furthermore, the high relative biological effectiveness of CIRT was observed to enhance tumor control while reducing overall toxicity. For example, a 92.6% two-year local control rate was achieved in hypofractionated HCC cohorts [10]. To contextualize these outcomes, historical data from a meta-analysis by Mizumoto et al. [13] reported substantially lower two-year overall survival rates for conventional photon modalities, specifically 32.6% for SBRT and 16.6% for 3D conformal radiotherapy (3D-CRT) in similar patient populations. This sharp contrast underscores CIRT's dosimetric superiority and its capacity for robust tumor eradication. However, as an exception, one suspected case of radiation-induced liver disease presenting with ascites was documented at 4.3 months post-CIRT in an intrahepatic cholangiocarcinoma patient [2,13].

Comparisons with photon-based studies further highlighted the dosimetric superiority of CIRT. In a cohort utilizing VMAT, radiation-induced liver disease was observed in 28% of patients receiving 50.4 Gy in 28 fractions. This toxicity was significantly associated with a larger planning target volume (620.2 cc in affected patients vs. 579.7 cc in unaffected patients; p = 0.028) [6]. This discrepancy in toxicity profiles was explained by the particle-specific Bragg peak, which contrasts sharply with the broader dose distribution characteristic of photon therapy. Notably, despite these dosimetric insights, no mechanistic details regarding biliary injury pathways, such as endothelial damage at the cellular level, were reported across the reviewed literature. Furthermore, the delayed nature of biliary injury may reflect the microvascular vulnerability of the peribiliary plexus, which is known to be susceptible to late radiation-induced ischemia and fibrosis. This mechanism has been described in photon-based hepatobiliary irradiation and may partially explain the delayed presentation of strictures observed after CIRT.

Summary of Evidence

Table 2 presents a summary of the evidence.

**Table 2:** Summary of evidence In the absence of a formal risk-of-bias tool (e.g., ROBINS-I) due to the narrative design of this review, "Confidence Level" represents a qualitative assessment of evidence strength. This level (e.g., high, moderate, low) was evaluated based on the rigor of the study design, sample size, and the overall consistency of the findings across the synthesized literature HCC: hepatocellular carcinoma; RBE: relative biological effectiveness; OS: overall survival

Theme	Key finding	Population applicability	Effect direction	Confidence level	Supporting studies
Incidence and timing	11% incidence (5/47) at median 8 months; grade 3 stenosis in 1/56	HCC and cholangiocarcinoma	Positive (complication observed)	Moderate	[2,8,11]
Risk factors	Perihilar HCC, macrovascular invasion, biliary V10% ≥ 40 Gy (RBE)	Primarily HCC	Positive (risks identified)	Moderate	[8,11]
Management and outcomes	Stenting in 3/5 severe cases; 23% deterioration, 68% 2-year OS	HCC with Child-Pugh B	Mixed (manageable but impacts function)	Limited	[11]
Dosimetric advantages	No strictures in syntheses; 92.6% 2-year local control	HCC and solid tumors	Negative (risk mitigation)	Strong	[1,5,10]

Discussion

Principal Findings and Their Interpretation

Our synthesis reveals that intrahepatic biliary stricture following CIRT represents an infrequent but clinically significant late toxicity. We hypothesize that this complication is primarily driven by dosimetric and anatomical factors. In this context, CIRT's inherent precision offers a protective edge over conventional modalities. The carbon ions' Bragg peak, in particular, enables steep dose gradients, concentrating energy at the tumor while limiting radiation spillover to the biliary endothelium. We contrast this favorable profile with photon therapy, where diffuse radiation exposure promotes fibrosis via chronic inflammation and vascular occlusion. However, we note that the reviewed studies only imply these mechanisms; they currently lack direct histological or biomarker data regarding pathways such as oxidative stress or TGF-β signaling in bile duct cells, underscoring the inferential nature of these pathophysiological assumptions.

When viewing the clinical data collectively, we find that the low incidence of strictures, such as the 11% observed in Child-Pugh B cohorts [11] across hypofractionated regimens, underscores a distinct dosimetric threshold effect. We suggest that limiting doses to biliary V10% < 40 Gy (RBE) may reduce the risk of overt strictures [8,11]. Crucially, as this specific dosimetric threshold is derived from limited retrospective evidence, it must be interpreted with clinical caution strictly as a hypothesis-generating concept, rather than a definitively validated universal guideline. This finding highlights CIRT's suitability for perihilar tumors, which clinicians typically deem high-risk for conventional photon approaches, provided these dosimetric constraints are carefully navigated.

While we suggest CIRT's dosimetric advantages based on consistent physical evidence across multiple prospective and retrospective designs, we interpret the reported incidence rates more cautiously. In strict alignment with our stratified synthesis approach, we recognize that proxy studies involving cholangiocarcinoma introduce inherent variability due to differing tumor biology; for instance, more infiltrative growth patterns potentially heighten ductal involvement independently of radiation. Ultimately, this hierarchy of evidence reflects robust physical and dosimetric data outweighing the currently sparse clinical complication reports. We note that CIRT demonstrates profound biological effectiveness against hypoxic HCC regions without inflicting an equivalent biliary penalty. Nevertheless, the absence of direct histological or biomarker data means that pathophysiological causality cannot yet be established beyond dosimetric correlations.

Comparison With Existing Literature and Resolution of Contradictions

Our findings align with the broader particle therapy literature. We observe that CIRT's high relative biological effectiveness yields superior local control (e.g., 92.6% at two years) compared to 3D-CRT or SBRT in meta-analyses of intrahepatic cholangiocarcinoma [13]. We attribute this superiority to enhanced DNA double-strand breaks in tumor cells, combined with the sparing of normal ductal epithelium via range modulation. This consistency across Japanese multicenter data and global reviews supports the generalizability of these findings [2,7,13,14].

However, we note specific contradictions in the literature: prospective hypofractionated trials report null biliary toxicities [10], whereas retrospective studies of Child-Pugh B cohorts signal positive stricture events [11]. We argue that this discrepancy likely stems from population heterogeneity. Specifically, impaired baseline liver function in Child-Pugh B patients amplifies radiosensitivity due to their reduced regenerative capacity. Furthermore, we suspect that healthier cohorts, which often exclude patients with macrovascular invasion, combined with shorter follow-up periods (e.g., 19 months in oligometastatic studies versus 25 months in primary HCC trials), lead to the under-detection of late-onset strictures [10,14].

We could not substantiate a definitive explanation for the dosimetric nulls observed in pancreatic proxy studies. In these cohorts, gastrointestinal toxicities dominated without a biliary focus, which we believe possibly reflects a selection bias toward non-hepatobiliary endpoints or residual confounding from re-irradiation intervals (e.g., a median of 15.8 months) [16]. Finally, we assess the risk of publication bias as moderate. Because positive efficacy outcomes dominate the literature (e.g., 85% one-year survival in oligometastatic disease), facilities prioritizing CIRT expansion might potentially underreport rare strictures [14]. Nevertheless, we maintain that the methodological evolution from early-phase trials in 2005 [9] to recent multicenter registries in 2024 [13] strengthens our confidence in the low-toxicity claims via improved treatment planning. When contrasted with earlier photon comparisons (e.g., a 28% incidence of radiation-induced liver disease in VMAT) [6], we highlight that CIRT's adoption effectively resolved prior over-toxicity issues.

Practical Implications

Based on our findings, we propose several practical implications for the clinical management of patients undergoing CIRT. To translate these findings into clinical practice, we have structured a proposed workflow for managing hepatobiliary risks (Table 3). For patients with unresectable perihilar HCC and macrovascular invasion-a demographic facing an 11% stricture risk-we recommend that clinicians prioritize specific preventive strategies. First, regarding dosimetric constraints, clinicians should consider restricting the biliary dose to biliary V10% < 40 Gy (RBE); however, we reiterate that this proposed constraint must currently be interpreted strictly as a hypothesis-generating concept rather than a validated universal threshold. Second, for imaging surveillance, we advise implementing regular monitoring at 8 to 12 months post-treatment to detect early signs of biliary dilation. Finally, we recommend a stepwise management approach, initially advising conservative care and reserving stenting primarily for cholangitis-prone cases, particularly in Child-Pugh B individuals, where a 23% risk of liver function decline warrants multidisciplinary hepatology input.

**Table 3:** Proposed clinical workflow for mitigating biliary stricture risk in CIRT The dosimetric threshold provided (V10% < 40 Gy (RBE)) is derived from limited retrospective data and is intended as a hypothesis-generating clinical reference rather than a definitive absolute constraint CIRT: carbon-ion radiotherapy; RBE: relative biological effectiveness; V10%: the percentage of the biliary volume receiving the specified dose

Phase	Category	Description
Phase 1: pre-treatment planning	Action	Identify high-risk anatomical features (e.g., perihilar tumors, macrovascular invasion) and baseline hepatic impairment (e.g., Child-Pugh B)
	Dosimetric Consideration	Consider restricting biliary V10% to < 40 Gy (RBE), treating this threshold strictly as a hypothesis-generating target rather than an absolute limit
Phase 2: post-treatment surveillance	Action	Implement extended imaging and biochemical follow-up (8-12 months and beyond), specifically targeting late-onset biliary dilation or elevated liver enzymes
	Management	Initiate conservative therapy for mild cases; escalate to percutaneous drainage or stenting promptly if refractory cholangitis or severe stricture develops

From a public health and clinical policy perspective, we acknowledge that the current evidence base, derived from relatively small cohorts, precludes the immediate integration of these specific dosimetric constraints into broad treatment guidelines. Instead, we suggest that specialized high-volume centers in high-incidence regions like Asia carefully consider these parameters on a case-by-case basis. We highlight that for patients refusing ablation, tailored follow-up protocols should be managed by a multidisciplinary hepatology team to prevent cholestasis-related hospitalizations. Furthermore, while regulatory bodies might explore expanded CIRT access for radioresistant tumors, it is recommended that such expansion be coupled with mandatory registry reporting to build a more robust safety profile.

We must, however, note important caveats regarding these implications. We base our recommendations primarily on Japanese HCC data, which we consider insufficient for direct application to Western or pediatric populations. Additionally, in alignment with our stratified synthesis approach, we caution that relying on proxy findings from pancreatic studies may overstate generalizability without further liver-specific validation.

Strengths and limitations

We consider the comprehensive database search, which successfully captured diverse study designs published between 2005 and 2025, to be a primary strength of this review. Additionally, our thematic synthesis effectively prioritizes biliary outcomes within a largely sparse and fragmented literature base. However, we must acknowledge several limitations within the included studies themselves. First, we note the inherent retrospective biases present in the majority of the analyzed cohorts. Second, the studies exhibited highly variable follow-up periods, ranging from 14.8 to 55.1 months, which complicates our longitudinal risk assessment. Third, while the inclusion of proxy populations, such as patients with pancreatic cancer, inherently introduces variability, we actively mitigated this by strictly applying our stratified synthesis approach to prevent the dilution of primary HCC findings. Finally, the reviewed literature completely lacks mechanistic assays to confirm the biological pathways of radiation-induced biliary injury.

Furthermore, we recognize specific limitations inherent to our review methodology. Regarding the literature search, although we did not conduct native queries directly within the PubMed or MEDLINE interfaces, we utilized Semantic Scholar and OpenAlex. Because these comprehensive academic graph databases automatically ingest and fully index primary biomedical records from PubMed and MEDLINE, supplemented by our manual cross-referencing of key articles, we maintain that our search strategy ensured robust and redundant coverage. Additionally, although we did not employ a formal risk-of-bias assessment tool (e.g., ROBINS-I or Cochrane RoB 2) due to the narrative design, we actively mitigated this shortcoming by conducting a rigorous qualitative confidence assessment across the synthesized themes. Finally, we acknowledge that a recent case report authored by the present investigator [23] was included to illustrate fatal infectious cascades. While this introduces a potential risk of self-citation bias, it was deemed clinically necessary due to the extreme rarity of such documented outcomes. Crucially, this report was utilized strictly as anecdotal evidence and was deliberately excluded from all primary incidence calculations to maintain strict statistical objectivity.

Gaps and future directions

We identified critical evidence gaps within the current literature. First, inconsistent biliary outcome definitions-such as relying on imaging versus biochemical confirmation-across studies substantially hinder direct comparability. Second, we note a significant underrepresentation of long-term (> 5 years) stricture incidence. Because most follow-up periods end at two to four years, studies are potentially missing the development of progressive fibrosis, particularly in compensated Child-Pugh A patients. Furthermore, we found a complete absence of mechanistic data regarding radiation-induced ductal pathways, such as endothelial apoptosis or periductal inflammation. This absence leaves observed dosimetric correlations biologically unvalidated. Additionally, proxy populations heavily dominate non-HCC contexts, and we currently lack direct CIRT data for vulnerable populations, such as those with transplanted livers, beyond isolated single-case reports [20].

To address these gaps, future prospective trials are essential to enroll primary HCC cohorts and mandate standardized CT or MRI surveillance at 6-24 months post-CIRT. Furthermore, we encourage researchers to incorporate specific dosimetric constraints (e.g., biliary V10% < 40 Gy (RBE)) and biomarkers (e.g., alkaline phosphatase trends) to develop robust risk prediction models. Moreover, we suggest that integrating methodological advances, such as real-time tumor motion tracking, could further refine the safety of hypofractionation regimens by minimizing unintended exposure to moving biliary structures. Multicenter studies are also warranted in underrepresented regions (e.g., Europe and North America) to mitigate the current geographic bias towards Asian cohorts. Ultimately, we propose targeted research on re-irradiation scenarios to resolve the current null findings in short-interval cases, thereby clarifying the cumulative risks of ductal injury.

## Conclusions

Intrahepatic biliary stricture following CIRT for liver malignancies is an infrequent but clinically significant late toxicity, primarily associated with perihilar tumors, macrovascular invasion, and high biliary radiation doses. Although severe cases may require interventional management such as stenting, CIRT remains an effective treatment for unresectable liver disease, offering superior dosimetric precision with a generally acceptable biliary toxicity profile. However, clinicians must remain vigilant, as rare but severe ischemic strictures can precipitate fatal infectious cascades or liver failure. Adherence to dosimetric constraints, particularly considering a threshold of biliary V10% < 40 Gy (RBE) strictly as a hypothesis-generating reference, alongside prolonged imaging surveillance, may be utilized when planning treatment in high-risk anatomical settings to mitigate stricture risk. Nevertheless, the absence of direct histological or biomarker data means that pathophysiological causality cannot yet be established beyond dosimetric correlations. Future prospective studies enrolling primary HCC cohorts with standardized biliary outcome definitions are needed to elucidate the long-term safety profile of CIRT; in the interim, careful patient selection and multidisciplinary management remain essential.
